# Modern Contraceptive Utilization Among Reproductive Aged Women in Ethiopia: Mixed Effect Multi‐Level, Regression, Health Analysis With Health Survey of Mini‐EDHS 2019

**DOI:** 10.1002/hsr2.72405

**Published:** 2026-04-19

**Authors:** Fassikaw Kebede Bizuneh, Tsehay Kebede Bizuneh

**Affiliations:** ^1^ College of Health science Debre Markos University Debre Markos Ethiopia; ^2^ Faculty of Social Science Bahir Dar University Bahir Dar Ethiopia

**Keywords:** Ethiopia, family planning, reproductive age, women

## Abstract

**Background:**

The use of contraceptives is key to reducing unsafe abortions from unintended pregnancies and helping to improve maternal health and socioeconomic outcomes. It allows individuals to achieve their desired family size and timing effectively. Therefore, this study aimed to identify multi‐level factors for Modern Contraceptive use among Reproductive‐Age Women in Ethiopia.

**Methods:**

Data for this study were extracted from the nationally representative 2019 Ethiopian Mini Health Analysis with Health Survey of Mini‐EDHS 2019. The survey employed a two‐stage cluster sampling design: enumeration areas (EAs) were selected in the first stage, followed by household selection in the second stage. Data collection took place from March 21 to June 28, 2019. A mixed‐effects multilevel analysis was used to identify factors associated with modern contraceptive use.

**Result:**

A total of 8885 (Wt = 100) participants were included in the study, with a mean age of participants 27.5 years (SD ± 10.3). In Ethiopia, modern contraceptive utilization among reproductive‐age women was 23.8%. The prevalence was 90.79% for married women and 3.74% for never‐in‐union women. The most common methods were injectable 13.6% and implants 5.8%. In the final multi‐level logistic regression being woman aged 15–25 years (AOR = 4.91; 95% CI: 2.70–11.38), having children 13–24 months (AOR = 2.40; 95% CI: 2.50–25.6), married women (AOR = 7.90; 95% CI: 2.47–25.60), Completed high school (AOR = 1.65; 95% CI: 1.20–2.60), middle wealth index (AOR = 1.55; 95% CI: 1.15–1.98), urban resident (AOR = 1.69; 95% CI: 1.20–2.40), living in Amhara region (AOR = 2.74; 95% CI: 1.24–3.50), and mass media exposures (AOR = 1.69; 95% CI: 1.21–2.93) were associated with modern contraceptives usages. In contrast, residing in the Somali region decreased the likelihood by 88% (AOR = 0.22; 95% CI: 0.11–0.45) of using contraceptives.

**Conclusion:**

Modern contraceptive use among women of reproductive age in Ethiopia remains critically low, falling short of the target set for 2030. The variation in usage is significantly influenced by both individual and community‐level factors. To address this gap, the government should prioritize investments in education and financial empowerment to strengthen women's autonomy in family planning decision‐making.

**Abbreviations**
AIC: Akaike's information criterion
AOR: adjusted odds ratio
EAs: enumeration areas
EDHS: Ethiopian Demographic and Health Survey
ICC: Intra‐cluster correlation
MOR: median odds ratio
PCV: proportional change in variance.

## 1 Introduction

Globally, sexual and reproductive health is a significant public health issue for women in the reproductive age group. Women from low‐ and middle‐income countries (LMICs) have an increased risk of mortality due to pregnancy‐related complications [1, 2]. Utilizing family planning methods helps prevent high‐risk pregnancies and unsafe abortions, and it empowers individuals to achieve their desired family size and timing efficiently [3, 4]. For example, the utilization of family planning methods could prevent an estimated 218 million unintended pregnancies, 55 million spontaneous births, 138 million premature births, and 118,000 maternal deaths in 2012 [5, 6]. Therefore, the use of contraceptives is considered a safe and cost‐effective approach to prevent unwanted pregnancies [7].

Globally, modern contraceptive utilization stands at 56% with a significant variation of 62% in developed nations and 26% in sub‐Saharan African countries [8, 9, 10]. Unintended pregnancies are a major challenge in low‐ and middle‐income countries (LMICs) and make up 89% of global abortions [11], but also enhance maternal and child health during postpartum [12]. Previous findings suggest that close birth spacing within the first‐year postpartum raises double the risks of preterm births, undernutrition after birth, and maternal complications [13]. In Sub‐Saharan Africa, high rates of unintended pregnancies and unsafe abortions contribute to significant public health risks for women, with one maternal death per 150 unsafe abortion cases, and 90% of abortions are unsafe [14, 15]. According to the 2017 World Family Planning Report, proper information on modern contraceptives and addressing side effects can prevent 32% of maternal and 10% of childhood deaths [15]. Globally, around 290,000 women aged 15 to 49 died from pregnancy‐related issues in 2015. Sub‐Saharan Africa accounted for 65% (179,000) of these deaths [6, 11].

In developing countries, waiting 24 months after childbirth with modern contraceptives before conceiving can reduce under‐5 child mortality by 13%. Extending this waiting period to 36 months could result in a 25% decrease [16]. In Uganda, 47% of reproductive‐age women do not use modern contraceptives despite needing them, as indicated by population‐level surveys [17]. In Ethiopia, the government has implemented strategies involving the integrated provision of family planning services with antenatal and postnatal care, focusing on postpartum family planning at least for 2 years in alignment with the FP2030 framework, to increase the rate from 22% to 70% by 2030 [18, 19]. However, in Ethiopia, an umbrella review using 56,169 reproductive‐aged women revealed the pooled prevalence of unmet family planning was found to be 27.44% [20]. A systematic review of factors associated with unmet family planning needs revealed that age, number of children, employment status, residence, level of education, fear of side effects, and religious beliefs hinder the use of modern contraceptives [6, 21].

A recent report in Ethiopia indicated that 11.7 million women desire to prevent pregnancy; however, 39% of them lacked access to modern contraceptives [4, 22, 23]. The 2011 EDHS found 25.3% of women had unmet needs, with 16.3% for spacing and 9% for limiting, and Rural areas showed higher unmet needs. In 2016, 58% of married women aged 15–49 desired family planning; with 36% already using contraceptives, Addis Ababa had the lowest unmet need (11%), while Oromia had the highest (29%) [21, 24]. The Federal Ministry of Ethiopia has been working to enhance modern contraceptive use by integrating family planning services with antenatal and postnatal care, following FP2030 framework strategies with progression reported ranging from 0.1% in the Somali region to 52.3% in Addis Ababa [25, 26]. However, the scoping review and qualitative findings on the reason Barriers to modern contraceptive utilization indicated that religious/cultural beliefs, fear of side effects, lack of knowledge, fear of infertility, partner resistance, and social norms hindered the utilization of modern family planning [7].

Several previous studies have investigated the factors that influence the utilization of modern contraceptive methods in Ethiopia. These factors typically fall into categories such as socioeconomic, demographic, and cultural influences [3, 15, 27, 28, 29]. According to studies, factors such as women's education level [15], women's age [3, 29], marital status [3, 11] number of living children [3], religion, place of residence [4, 29], wealth index [3, 11], exposure to family planning messages [3], occupational status [3, 11] are associated with modern family planning utilization. Some studies have indicated that factors such as maternal health care services utilization, including antenatal visits [11], postnatal care utilization [5, 12, 13], and health facility delivery are associated with modern contraceptive use. In Ethiopia, previous study findings [3, 4, 6, 15, 25, 27, 29] indicate that modern contraceptive utilization predominantly centers around a limited set of modern contraceptive methods such as pills, IUDs, injections, condoms, and implants, with a primary focus on married women. However, this study included all sexually active women, including those who were never in a union, divorced, and widowed. Specific contraceptive methods such as male sterilization, lactation amenorrhea, and the Standard Days Method, which are widely accessible in Ethiopia, were included in this study. Conducting this research filled a crucial gap by considering a wide array of contraceptive methods and a diverse group of sexually active reproductive‐age women nationwide. The study aimed to identify multilevel factors for modern contraceptive utilization among reproductive‐age women in Ethiopia using data from the 2019 min‐Ethiopian Demographic and Health Survey.

## 2 Methods

### 2.1 Study Setting, and Data Source

**Figure 1:**
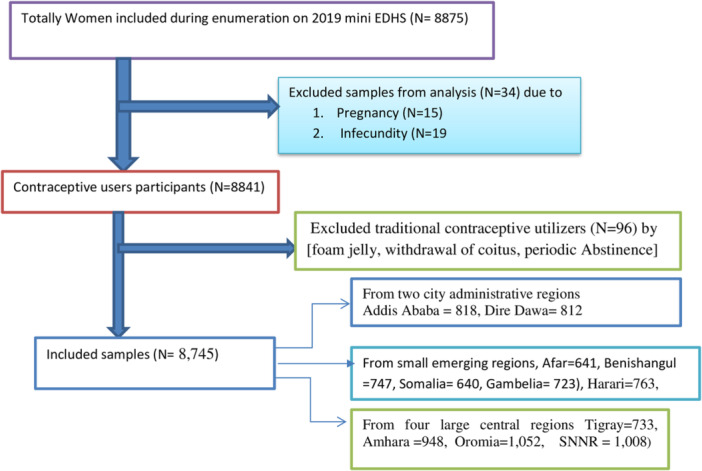
Schematic presentation for modern contraceptive use among reproductive‐aged women in Ethiopia.

For this study, we utilized the secondary dataset from the 2019 Ethiopia Mini Demographic and Health Survey (EMDHS), which was made available by the Central Statistical Agency of Ethiopia [30]. This survey marks the second EMDHS and the fifth Demographic and Health Survey conducted in Ethiopia. The primary aim of the survey was to offer current estimates for health and demographic variables of interest at both national and regional levels, including urban and rural areas. The survey was carried out from March 21, 2019, to June 28, 2019, based on a nationally representative sample [31]. The study was conducted in nine regional states and two administrative cities found in Ethiopia regional states in Ethiopia (Tigray, Afar, Amhara, Oromia, Benishangul, Gambela, South Nation, Nationalities and Peoples’ Region (SNNPR), Harari and Somali), and two administrative cities (Addis Ababa and Dire‐Dawa) conducted from 10 February 2019 to June 5, 2019 [32] (Figure 1).

### 2.2 Data Source and Sample Weighting

To get a dataset, we first submitted a research proposal for the DHS office to access the min EDHS 2019 dataset. In the 2019 EDHS, 9,150 households were chosen for the sample, with 9012 eligible women for interviewing participating in the survey and 8,885 reproductive‐age women providing complete information during data collection [32]. The DHS Program Office provided an authorization letter to access the 2019 EDHS, the fourth comprehensive survey. EDHS 2019 encompassed 9150 households across nine regions and two administrative divisions. A detailed protocol outlining the data collection process and sampling methodology employed in the EDHS 2019 is publicly available for review at www.measuredhs.com [31, 33]. We applied weighting to the sample by utilizing the individual weight assigned to women (hv005) to ensure a proper representation. The sample weights were calculated by dividing the individual weight (hv005) by 1,000,000.

### 2.3 Population and Sampling Procedures

The source population for this study consisted of all women in the reproductive age group between the ages of 15–49 years residing in the selected households, and who were present during the data collection period constituted the study population. All sexually active reproductive‐age women, regardless of marital status, were the source population for this study.

Each household served as the sampling unit, and each woman was treated as the study unit. The regions were divided into urban and rural clusters, resulting in a total of 21 sampling strata [30]. The EDHS 2019 utilized a multistage cluster sampling approach. In the initial stage, 305 Enumeration Areas (EAs) were randomly selected, with 93 in urban regions and 212 in rural regions, and were distributed proportionally based on household size across the sampling strata [30]. Subsequently, 30 households per cluster were selected using systematic random sampling with equal probabilities. Ultimately, a representative sample of 9150 households was selected for the 2019 EDHS, with 8794 households being occupied during data collection [3, 29]. In our study, 8745 female participants were included after excluding pregnant women from the analysis of eligible women between the ages of 15–49 years. A simple random sampling strategy was employed to choose one woman from households with multiple eligible women, whereas, before the analysis, pregnant women were excluded.

### 2.4 Data Collection Tool and Procedure

The EDHS employed a structured and pre‐tested questionnaire for data collection. Trained interviewers conducted structured interviews in the local language. The women's questionnaire gathered information on socio‐demographic, maternal family, and reproductive health‐related characteristics. In the 2019 EDHS, women of reproductive age were queried about their fertility intentions, past pregnancies, the number of children they have given birth to, marital status, including current pregnancy status, and the usage of various contraceptive types and methods.

### 2.5 Outcome Variable

The outcome variable in the study pertained to the utilization of modern contraceptive methods among Ethiopian women of reproductive age, which was dichotomously categorized as “Yes” or “No”. This classification was derived from responses to the query regarding the specific contraceptive approach being employed to prevent or postpone pregnancies. The available options included “no method,” “folkloric method,” “traditional method,” and “modern method”. Consequently, this led to the creation of a binary outcome variable, where individuals currently utilizing modern contraceptive techniques were categorized as “Yes,” while those not using such methods were categorized as “No”. For further insights into the various types and definitions of contraceptive methods, interested readers are encouraged to refer to additional resources [30].

**Independent variables:** during the final data analysis, two classes of independent variables were used to identify maternal individual and community‐level factors.

### 2.6 Individual‐Level Characteristics (Level I)

Which included maternal variables including maternal age (15–49), education level (no education, primary, secondary, and higher), religion (Protestant, Muslim, Catholic, Orthodox, and others), number of live children in the past 5 years, wealth index (Poor, middle, rich, and too rich), media exposure (yes, no), age of children, and family size of the participant.

### 2.7 Community‐Level Factors (Level II)

Which included maternal factors at the community level, such as residence (urban and rural) and media exposure related to family planning (Radio, TV, Newspaper, Mobile messages) categorized as (Yes/No). These community‐level variables provided insights into the broader social and environmental context affecting women in the community, contributing to a comprehensive analysis of factors influencing reproductive‐aged women in Ethiopia (Table 1).

**Table 1:** Independent variables for maternal modern contraceptive utilization among 15–48 years of women.

Variables	Categorization/operational words
Age of respondents	Categorized into three groups: 15–24, 25–34, 35–49
Educational status	Categorized as no formal education, primary education, secondary, and above
Wealth index	Poor, middle, rich
The household has television	Household television access was categorized as No and Yes
Household has radio	Household radio access was categorized as No and Yes
Number of under‐five children	Categorized as none, 1–4, 5–8, ≥ 9
Births in the last 3 years:	Categorized as no birth, one birth, two and more births
	Categorized as No, yes
Resident	Categorized as urban/rural.
Media exposure status	Categorized as Yes or No
Regions classification	Tigray, Afar, Amhara, Oromia, Benishangul, Gambela, South Nation, Nationalities and Peoples' Region (SNNPR), Harari, and Somali, and two cities (Addis Ababa and Dire‐Dawa)
Religion classification	Orthodox Christian, Muslim, Protestant, others

### 2.8 Operational Words

**Modern contraceptives:** encompass a variety of methods, including pills, IUDs, injections, diaphragms, implants, and female condoms, and male sterilization, except for traditional contraceptive practices methods, including foam‐jelly, coitus interrupts/or withdrawal methods for family planning purposes were considered for this research [34]. Sexually active reproductive age women typically refer to women who are of reproductive age (generally defined as being between the ages of 15 and 49 years old), regardless of marital status.

**Mass Media Exposure:** The Ethiopian Demographic and Health Survey (EDHS) asked women if they had been exposed to family planning messages through radio, television, print media (e.g., magazines and newspapers), or text messages a week before the interview. Due to the high correlation among these four variables, a new variable called ‘mass media exposure to family planning messages’ was created, and respondents were coded as “yes” if they reported being exposed to family planning messages through at least one of the four media outlets. Respondents were coded as “yes” if they reported being exposed to family planning messages through at least one of the four media outlets, and as “no” if they reported no exposure (coded as 0 for no, 1 for yes) [35].

### 2.9 Data Processing and Statistical Analysis

In this study, the statistical analysis was conducted using the statistical software STATA version 17.0. Both descriptive and inferential statistical methods were employed. To summarize the distribution of socioeconomic and demographic characteristics of the respondents and utilization of modern contraceptives, frequencies and proportions were utilized [30]. To ensure accurate inference and efficient parameter estimation, it is essential to consider this correlation by employing appropriate analysis methods. The mixed‐effect multi‐level modeling was used to accommodate the cluster‐level variation of study participants [36].

In this study, various nested models were employed, including a null model (Model 1) devoid of explanatory variables, Model II incorporating individual‐level variables, Model III integrating community‐level factors, and Model IV combining both individual and community‐level variables. During the modeling Building, the ICC was found to be ≥ 34.1%, indicating significant variation for modern contraceptive utilization among cluster levels. Therefore, we built consecutive nested multi‐level analyses, including individual‐level variables with outcome variables, and model III (community‐level variables with outcome variables).

Finally, we built model IV (All individual‐level and Community‐level factors with outcome variables) to get statistically significant variables. The evaluation of clustering effects involved computing the Intra‐class Correlation Coefficient (ICC) and Median Odds Ratio (MOR). The ICC was determined for each model using the formula ICC = variance of each model/(variance of each model + 3.29), while the MOR was calculated as MOR = exp(0.95 * cluster level variance) [3, 36]. During Model II and Model III bi‐variable multilevel logistic regression, identifying categorical variables associated with modern contraceptive utilization at *p* ≤ 0.25 were candidates transferred for the final model. Adjusted Odds Ratios (AOR) with 95% confidence intervals with a significance level of *p* < 0.05 were claimed significant factors for modern contraceptive utilization.

### 2.10 Model Adequacy Test

We compared and assessed the final model adequacy test for selection as the best model. The fitness of the model was evaluated using the Intra‐class Correlation Coefficient (ICC), Likelihood Ratio (LR) test, Median Odds Ratio (MOR), deviance (‐2LLR), Akaike's Information Criteria (AIC), and Bayesian Information Criteria (BIC) values by comparing their respective coefficients. The final model (Model III), which included both individual and community‐level variables, was found to be the best‐fit model for this study [34].

## 3 Result

### 3.1 Socio‐Demographic Characteristics of Participants

A total of 8745 individual women participated in this report. The mean age of participants was 27.5 (standard deviation ± 10.3) years. More than one in five respondents were aged 15–19 years (*n* = 2056, 23.6%) and 25 to 39 years. Out of the total respondents, 40.7% (3,247 of the women had not attended formal education; however, only 8.47% (675) of the participants had a diploma and above educational levels. The majority of 4495 (51.4%) participants were women, had 5 to 8 family members in a house, whereas nearly two in every five, 3329 (38.07%) of study participants were found under the poor wealth quintiles class (Table 2).

**Table 2:** Socio‐Demographic profiles of respondents for mass media exposure and contraceptive utilization of sexually active women in Ethiopia, Mini EDHS 2019.

Variable	Categories	Number (weighted)	Frequency
Age of respondent	15–25	4240	48.59
	26–35	2709	30.90
	36–45	1536	17.53
	≥ 46	260	2.97
Resident	Urban	2892	33.10
	Rural	5853	66.92
level of education	No education	3598	41.21
	Primary	3298	37.73
	Secondary	1117	12.80
	Higher	732	8.40
Wealth index	Poor	3329	38.07
	Middle	1251	14.31
	Rich	4165	47.63
Religion	Orthodox	3374	37.97
	Catholic	78	0.88
	Protestant	1711	19.26
	Muslim	3635	40.91
	Traditional	60	0.68
	Other	27	0.30
Mass media utilization	Yes	2647	30.27
	No	6098	69.73
Household members	≤ 4	3217	36.79
	5–8	4495	51.40
	≥ 9	1033	11.81
Number of living children	No children	3044	34.81
	1–4	3899	44.59
	5–8	1,673	19.13
	≥ 9	129	1.48
Current age of children (*N* = 5470)	Less than a month	2038	37.26
	2–12 months	3056	55.87
	13–24 months	347	6.34
	≥ 25	29	0.53
Knowledge of family planning	knows no method	643	7.35
	knows only the folkloric method	2	0.02
	knows only the traditional method	14	0.16
	knows the modern method	8086	92.46
Regions	Tigray	714	8.16
	Afar	636	7.27
	Amhara	926	10.59
	Oromia	1035	11.84
	Somalia	639	7.31
	SNNR	1000	11.44
	Benishangul	734	8.39
	Gambella	712	8.14
	Harari	747	8.54
	Addis Ababa	806	9.22
	Dire Dawa	796	9.10

### 3.2 Magnitudes of Modern Contraceptive Utilization

The overall magnitude of modern contraceptive utilization among reproductive‐aged women in Ethiopia was found to be 23.8% (95% CI: 22.9%–24.77%). This rate was notably higher among married women (90.79%) in comparison to women who had never been in the union. Across regions, modern contraceptive utilization varied, with lower rates observed in regions such as Ethiopia Somalia at 0.17%, and Afar at 0.72%, compared with Tigray at 2.07%, Amhara at 3.55%, SNNPR at 3.62%, Oromia at 3.42%, Benishangul‐Gumuz at 2.39%, Addis Ababa at 2.44%, Gambela at 2.01%, Harari at 1.93%, and Dire Dawa at 1.65%.

### 3.3 The Measure of Cluster‐Level Variation

During the null model of multi‐level regression, the ICC was 34.1% of the variations of modern contraceptive utilization among study subjects were attributed to differences at the cluster level, whereas the remaining 65.9% were attributed to individual women's factors. Likewise, in Model II and Model III, the ICCs were estimated to be 18.53% and 10.95%, respectively, both individual influence, which indicates modern contraceptive use and community‐level factors in every nine regions, and had significant level variation. In the final model (Model IV) of the multi‐level regression, the ICC was estimated as 15.4%, indicating that 15.4% of the total variation in modern contraceptive usage among women can be attributed to both individual‐level and community‐level factors. Additionally, the proportional change in variance value of 0.59% suggests that these factors explain 59% of the cluster‐level variation in modern contraceptive utilization among sexually active women. However, 40.6% of the cluster‐level variation remains unexplained, possibly due to unmeasured factors or variability (Table 3).

**Table 3:** Random effect analysis and model fitness for factors associated with modern contraceptive utilization among reproductive‐aged women in Ethiopia min‐EDHS 2019.

	Model I	Model II	Model III	Model IV
**Radom effect**				
Community variance	0.628 (0.09)*	0.726 (0.133)*	0.43 (0.0649)*	0.59 (0.108)*
ICC	15.20 (12.21–20.50)	18.50 (13.31–24.10)	10.9 (8.2–14.70)	15.4 (11.4–20.5)
PCV	Reference	51.26%	67.89%	53.72%
MOD	2.12	1.37	1.272	1.366
**Model fitness**				
Log likelihood	−4975.35	−3041.66	−4912.07	−2998.53
AIC	9954.70	6143.32	9850.15	6079.03
BIC	9968.85	6341.53	9942.14	6349.92

*Indicate the significant of the associated variabels.

### 3.4 Multi‐Level Associated Factors

In the final model (Model IV), both individual and community‐level factors were associated with modern contraceptive utilization for reproductive‐age women. Accordingly, a reproductive‐aged woman 15–25 years had four (AOR = 4.91, 95%CI: 27–11.38, *p* = 0.001) times higher likelihood of utilizing modern contraceptives as compared to women aged ≥ 49 years.

Likewise, the adjusted odds ratio (AOR) for women having children aged between 13 and 24 months had two (AOR = 2.4) higher likelihood of contraceptive usage compared to women who had never been married (95% CI: 2.5–25.6). Moreover, the odds of utilizing modern contraception among married women in Ethiopia were found eight (AOR = 7.9) times higher compared to women who were never in union [95% CI: 2.47–25.6], associated with a higher likelihood of planning to use modern contraception. The study found that reproductive‐age women with higher education levels had a twofold (AOR = 1.65) increased likelihood of planning to use modern contraception compared to their counterparts (95% CI: 1.20–2.6). Women in Ethiopia from households with a middle wealth index had two (AOR = 1.55) higher odds of planning to utilize contraceptives compared to those from poor wealth index households (95% CI: 1.15–1.98). Moreover, reproductive‐age women residing in urban areas were twofold (AOR = 1.69) times more likely to use modern contraceptives compared to those living in rural areas (95% CI: 1.2–2.4).

Reproductive‐aged women in Ethiopia and Somalia regions had an 88%(AOR = 0.22) lower likelihood of using modern contraceptives (95% CI: 0.11–0.45), whereas women living in the Amhara region had three times (AOR = 2.74) increased the likelihood of using modern contraceptives compared to Dire‐Dawa (95% CI: 1.24–3.50). Furthermore, previous mass media exposure on family planning was found to be associated with nearly a twofold (AOR = 1.69) increase in the odds of utilizing modern contraceptives among reproductive‐age women in Ethiopia, compared to the controls (95% CI: 1.21–2.93) (Table 4).

**Table 4:** Multivariable multilevel analysis for determinants of modern contraceptive utilization of sexually active reproductive women in Ethiopia, data from Mini‐2019 EDHS.

Variables	Categories	Model I	Model II AOR (95%CI)	Model III AOR (95% CI)	Model IV AOR (95% CI)
Age of women	≤ 25		4.61 [2.12–10.87]***		4.91 [2.27–11.38]***
	26–35		3.32 [1.8–8.81]**		2.29 [0.98–8.75]
	36–45		2.60 [1.20–5.83]*		1.16 [0.92–7.86]
	≥ 46		Ref		Ref
Marital status	Never in Union		Ref		Ref
	Married		44.5 [28.33–70.25]***		7.93 [2.47–25.6]**
	Divorced		50.1 [24.20– 104.27]***		1.90 [0.93–17.]
	Living with partners		0.99 [0.28–3.54]		0.37 [0.12–1.62]
	Separated		7.85 [3.22–15.74]***		1.08 [0.3– 3.9]
	Separated		5.19 [2.6–13.64]		0.41 [0.13–1.56]
Level of education	No education		Ref		Ref
	Primary		1.54 [1.17–2.03]**		1.46 [0.81–1.6]
	Secondary		1.71 [1.14–2.60]**		1.65 [1.20–2.59]***
	Higher		1.47 [0.7– 3.1]		1.41 [0.67–3.13]
Wealth index	Poor		Ref		Ref
	Middle		1.69 [1.24– 2.14]**		1.55 [1.15–1.98]*
	Rich		1.40 [1.02–1.78]*		1.27 [0.89–1.60]
Birth in the last 3 years	No child		Ref		Ref
	1		0.97 [0.71–1.34]		0.97 [0.71–1.49]
	≥ 2		0.96 [0.55–1.7]		1.12 [0.56–1.85]
Age of children	≤ 30 days		2.71 [1.31–17.40]**		1.63 [0.96–17.74]
	2–12 months		3.88 [1.98–12.4)*		1.34 [0.92–1.81]
	13–24 months		2.62 [1.82–5.7]*		2.41 [1.35–5.42]**
	≥ 25		Ref		Ref
Regions	Tigray			1.78 [1.23–2.6]**	1.15 [0.66–1.91]
	Afar			0.69 [0.33–1.12]	1.1 [0.49–1.65]
	Amhara			2.70 [1.86–3.9]**	2.74 [1.24–3.50]*
	Oromia			2.10 [1.48–3.2]**	1.7 [0.98– 2.91]
	Somalia			0.11 [0.14–0.34]*	0.12 [0.11–0.45]***
	SNNR			2.56 [1.72–3.76]**	2.05 [0.92–3.5]
	Benishangul			2.02 [1.37–2.97]**	1.61 [1.92–2.55]
	Gambella			1.12 [0.83–2.77]	0.91 [0.44–1.93]
	Harari			1.19 [0.86–1.65]	0.98 [0.65–1.30]
	Addis Ababa			1.50 [1.14–2.13]**	1.55 [0.99–2.31]
	Dire Dawa			Ref	Ref
Resident	Rural			Ref	Ref
	Urban			1.41 [1.12–1.78]**	1.69 [1.21–2.93]*
Mass media exposure	Yes			1.36 [1.24–1.73]**	1.22 [1.12–1.93]*
	No			Ref	Ref

* *p* < 0.05

** *p* < 0.001

*** *p* < 0.0001.

## 4 Discussion

This study aimed to correlate modern contraceptive utilization with multi‐level factors among women of reproductive age, including those who are divorced, never in a union, separated, widowed, and married, in Ethiopia. In the final report, the overall prevalence of modern contraceptive use among sexually active women of reproductive age (including those who are married, widowed, never in a union, and separated) was found to be 23.8%. This result is consistent with 22.2% previously reported findings in Ethiopia [37], and pooled results in 20.68% of ten Sub‐Saharan African countries (Ethiopia, Kenya, Comoros Malawi, Mozambique, Rwanda, Tanzania, Uganda, Zambia, and Zimbabwe) [34] and the finding was higher than previously reported 8.5% in Afar regions, Eater Ethiopia [38]. The possible reason for the estimated differences could be due to participants’ cultural, religious, and awareness differences for contraceptive usage. Conversely, our finding is lower than studies in the Amhara region 42.3% [3], and 55% finding in India [39]. The dissimilarity in results may be due to differences in study design, sample sizes, as well as social pressures and geographical variations.

In this study, the usage of contraceptives was strongly related to women of reproductive age. Consistent with previous research [31, 34, 40, 41], being a woman aged 15–25 years was significantly associated with a higher likelihood of modern contraceptive usage. This may be because younger women (15–24 years) perceive higher risk behavior due to early sexual activity, coupled with a strong intention to use early‐age family planning [42]. The use of modern contraceptive methods was found to be associated with the age of the children. Women who had children aged between 13 and 24 months had twice the likelihood of using contraceptives compared to women with children aged 25 months or older. This finding is consistent with previous research conducted in Ethiopia [40, 43, 44, 45, 46, 47], findings reported in Addis Ababa [48], and Nigeria [49]. This might be due to women being motivated to use modern contraceptives after childbirth, especially within the first 2 years, to address concerns about closely spaced pregnancies and to control family size by delaying or spacing subsequent pregnancies.

The report of this study indicated that married women were nearly eight times more likely to increase the odds of utilizing contraceptives compared with those who had never been in a union. This is consistent with previous study findings reported in Ethiopia [2, 50] that married women had nearly eight times more likely increased odds of utilizing contraceptives compared with women who had never been in a union. The possible reason might be that marriage likely broadens women's social connections and resources, which enhances access to media resources through social networks and joins with decision‐making with their husbands for the utilization of contraceptive methods.

The final report of this study indicated women found in the Somali region had a 12.1% lower likelihood of utilizing modern contraceptives [31, 51]. On the other hand, two‐fold increase in the utilization of contraceptives for reproductive‐age women living in the Amara region compared with control groups. This report is consistent with 2016 DHS findings [50, 51]. The variation in contraceptive utilization across regions can be attributed to cultural and geographical differences, influencing estimation variations within the country. In line with reported results in Addis Ababa [48] and Nigeria [49], the age of children was found to be a significant factor for initiating contraceptive use for sexually active women after birth.

Sexually active women who completed secondary education had twice the likelihood of using modern contraceptives compared to those with no formal education. This finding is consistent with previously reported results in Ethiopia [52], Nigeria [34], and 26 SSA countries (Including Angola, Burundi, Chad, DR Congo, Rwanda, Ethiopia, Kenya, Tanzania, Uganda, Lesotho, Malawi, Namibia, South Africa, Zambia, Zimbabwe, Benin, Cameroon, Gambia, Ghana, Guinea, Liberia, Mali, Nigeria, Senegal, Sierra Leone, and Togo in 2019) was 20.4% [35]. This could be attributed to the positive impacts of education on reproductive health, as it enhances decision‐making abilities, enables informed choices, and increases access to media promoting modern contraceptives.

The final report of the study indicated women with a middle wealth index in a house had double‐fold increased utilization of modern contraceptives compared with poor wealth index. This was supported by previous findings reported in Ethiopia [31, 34, 51, 53], results in Nigeria [49], and findings reported in India [54]. This could be attributed to individuals’ ability to afford modern contraceptives independently, without relying solely on their partners, since wealth index assets, including cell phones, radios, televisions, and cars, provide access to updated information through mass media platforms and enable decision‐making on reproductive health. Inversely, women in the poorest wealth index face challenges in acquiring such gadgets due to high costs. In line with previous findings of SSA [35] and the Philippines [55], there is a positive association of contraceptive use with women's previous mass media exposure for family planning. This might be due to the fact that mass media have the potential to influence contraceptive behaviors by stimulating individuals to adopt independent contraceptive usage. Moreover, women in urban parts of Ethiopia had a double‐fold increased likelihood of using modern contraceptives, as compared to those residing in rural settings. This is consistent with previous findings in Ethiopia [31] and Burkina Faso [56]. This might be related to women from urban areas being highly exposed to mass media (TV and radio), which helps for better access, prioritizing structural issues, and providing the best reproductive health service for women.

### 4.1 Limitations of the Study

This study utilized the most recent nationally representative data collected with validated and standardized tools and employed robust modeling to consider cluster‐level variation for modern contraceptive use. It also utilized multilevel analysis to adjust for the correlated nature of the EDHS data. However, essential factors such as women's attitudes, knowledge about contraceptives, and partner perspectives were not included. Moreover, assuming constant predictor effects across all levels and the potential for bias due to unobserved heterogeneity across groups are limitations of the study.

## 5 Conclusion

Modern contraceptive use among women of reproductive age in Ethiopia remains critically low, falling short of the target set for 2030. The variation in usage is significantly influenced by both individual and community‐level factors. To address this gap, the government should prioritize investments in education and financial empowerment to strengthen women's autonomy in family planning decision‐making.

## Author Contributions

**Fassikaw Kebede Bizuneh:** contributed to all major aspects of this work, including conceptualization, investigation, funding acquisition, methodology, validation, visualization, software, formal analysis, project administration, data curation, supervision, and resources. As the first author, he led the study design, performed all technical analyses, developed the software, curated the data, created all visualizations, and oversaw the entire research process from inception to completion. **Tsehay Kebede Bizuneh:** provided supporting contributions in writing – review and editing, as well as assisting with validation and investigation where needed. While the second author played a collaborative role, the primary intellectual and technical workload, along with project leadership and resource management, was carried out by the first author.

## Funding

The authors have nothing to report.

## Ethics Statement

This study used publicly available secondary data from the Demographic and Health Survey (DHS) repository, accessible freely online after submitting a project abstract for the women's concern code of IR to the Mini‐2019 EDHS dataset. The dataset, including data from the women's questionnaire and some household variables, is accessible on the Measure DHS website at https://dhsprogram.com. Ethical clearance for the study was obtained from the Ethiopian Public Health Institute (EPHI) and the National Research Review Board, and written informed consent was obtained from all participating women.

## Consent

The authors have nothing to report.

## Conflicts of Interest

The authors declare no conflicts of interest.

## Transparency Statement

The lead author, Fassikaw Kebede Bizuneh, affirms that this manuscript is an honest, accurate, and transparent account of the study being reported; that no important aspects of the study have been omitted; and that any discrepancies from the study as planned (and, if relevant, registered) have been explained.

## Data Availability

The datasets generated and/or analyzed during the current study are available from the corresponding author on reasonable request.
